# It’s not you versus us, it’s us versus the virus

**DOI:** 10.1017/ice.2020.258

**Published:** 2020-05-27

**Authors:** Hitoshi Honda

**Affiliations:** Division of Infectious Diseases, Tokyo Metropolitan Tama Medical Center, Tokyo, Japan

*To the Editor*—The cluster of novel coronavirus disease 2019 (COVID-19) originating in Wuhan, China, has now exploded into a pandemic via community spread. The COVID-19 epidemic in Japan began sometime between January and mid-February 2020. It is thought to have been brought to Japan by evacuees from Wuhan, China, or by infected passengers aboard a cruise ship. These patients were admitted to hospitals designated by the Japanese government for the treatment and containment of specific infectious diseases. However, these hospitals were unable to cope with the mounting numbers of patients, some of whom were referred to other tertiary-care centers. With the spread of the disease, a number of healthcare workers (HCWs) became infected with COVID-19, and their identity was made public by the Japanese mass media in reports carrying nuances of blame directed at the affected HCWs for having become infected. These media cited inadequate preparedness or insufficient infection control measures at hospitals as grounds for the blame they directed at the affected HCWs and continue to strongly influenced public opinion about the COVID-19 pandemic.

One of the first outbreaks among HCWs in Japan occurred among a group of resident physicians who held a social gathering.^1^ As infections have continue to spread throughout the community, some hospitals have reported instances of nosocomial transmission between HCWs, especially at facilities that admitted patients with COVID-19 in the early stages of the pandemic. These facts were taken up by the media in the manner described above, undermining the reputation of HCWs in general who daily put their own health at risk to provide care to patients with COVID-19.

In the wake of such episodes, hospital administrations have been obliged to issue press releases or hold press conferences whenever any of their healthcare staff tested positive or cases of nosocomial transmission of SARS-CoV2 were confirmed within their facility. Such announcements have typically included an apology on behalf of the sick HCWs and for possible in-facility transmission of the virus. As a consequence of the negative press, some hospitals have resorted to removing exposed HCWs from the frontline until they are cleared of COVID-19, hampering efforts to treat patients and control the spread of infections. Furthermore, these hospitals have sometimes reacted to public scrutiny by restricting or intermittently terminating the admission of newly infected patients to contain the nosocomial spread of COVID-19 despite the urgent need to treat more, rather than fewer, patients as the pandemic continues to spread unabated.

Outside the hospital setting, Japan has witnessed instances of discrimination against HCWs who may have had close contact with patients with confirmed or suspected COVID-19; both HCWs and their families have been slandered, and in some cases their children have been barred from entering nurseries or schools. Similar episodes of harassment or stigmatization of HCWs and their families have been reported from Singapore, Philippines, and other Asian nations as well as from Australia and the United States.^2-4^

Healthcare workers worldwide are at much higher risk of contracting COVID-19 by virtue of the work they do. Indeed, the number of HCWs who have become critically ill or have died from COVID-19 continues to mount daily.^5^ Unfortunately, breaches in infection control can and do occur, given the dimensions, rapidity, and severity of the current pandemic, for which no government or facility was adequately prepared. The use of bulky equipment such as extracorporeal membrane oxygenation has made zoning difficult in the nosocomial setting. Airway management, including endotracheal intubation, requires considerable preparation prior to the procedure. In addition to these difficulties, HCWs also face shortages of personal protective equipment, which exponentially increase the risk of exposure to COVID-19 during patient care.

Moreover, the potential for SARS-CoV2 transmission from asymptomatic or minimally symptomatic patients has exacerbated the situation for HCWs.^6^ Anecdotal reports have described cases of patients receiving treatment for an unrelated illness during hospitalization, only to be found later to have been harboring SARS-CoV-2 unbeknownst to the HCWs. Also, as with the rest of the public, healthcare workers themselves are susceptible to community-acquired COVID-19 and may unwittingly carry the virus into the hospitals where they work if they are asymptomatic or minimally symptomatic.

A public mindset of zero tolerance for COVID-19 among HCWs exerts powerful and deleterious pressure on the healthcare system. An attitude of zero tolerance is fueled by the public’s understandable fear of the spread of the disease but ultimately stems from an irrational response that can have only negative consequences for society in general. Criticizing HCWs for having contracted COVID-19 merely dampens their motivation to do their duty, increases their stress levels, and leads to ad hoc policies that set back treatment facilities in their fight against COVID-19.

Improving the public’s understanding of COVID-19, in terms of its ease and modes of transmission and the numerous challenges faced by HCWs in battling the disease, is as equally important as effective political leadership in this time of global crisis—not only for the men and women in the frontline of the war against this pathogen but also, by extension, for the well-being of society at large.
